# Back to the Future: From Appendage Development Toward Future Human Hair Follicle Neogenesis

**DOI:** 10.3389/fcell.2021.661787

**Published:** 2021-04-12

**Authors:** Simon C. de Groot, Magda M. W. Ulrich, Coen G. Gho, Margriet A. Huisman

**Affiliations:** ^1^Association of Dutch Burn Centres, Beverwijk, Netherlands; ^2^Hair Science Institute, Maastricht, Netherlands; ^3^Department of Otorhinolaryngology and Head & Neck Surgery, Leiden University Medical Center, Leiden, Netherlands

**Keywords:** hair follicle, neogenesis, organogenesis, alopecia, extracellular matrix, tissue engineering, hair follicle development

## Abstract

Hair disorders such as alopecia and hirsutism often impact the social and psychological well-being of an individual. This also holds true for patients with severe burns who have lost their hair follicles (HFs). HFs stimulate proper wound healing and prevent scar formation; thus, HF research can benefit numerous patients. Although hair development and hair disorders are intensively studied, human HF development has not been fully elucidated. Research on human fetal material is often subject to restrictions, and thus development, disease, and wound healing studies remain largely dependent on time-consuming and costly animal studies. Although animal experiments have yielded considerable and useful information, it is increasingly recognized that significant differences exist between animal and human skin and that it is important to obtain meaningful human models. Human disease specific models could therefore play a key role in future therapy. To this end, hair organoids or hair-bearing skin-on-chip created from the patient’s own cells can be used. To create such a complex 3D structure, knowledge of hair genesis, i.e., the early developmental process, is indispensable. Thus, uncovering the mechanisms underlying how HF progenitor cells within human fetal skin form hair buds and subsequently HFs is of interest. Organoid studies have shown that nearly all organs can be recapitulated as mini-organs by mimicking embryonic conditions and utilizing the relevant morphogens and extracellular matrix (ECM) proteins. Therefore, knowledge of the cellular and ECM proteins in the skin of human fetuses is critical to understand the evolution of epithelial tissues, including skin appendages. This review aims to provide an overview of our current understanding of the cellular changes occurring during human skin and HF development. We further discuss the potential implementation of this knowledge in establishing a human *in vitro* model of a full skin substitute containing hair follicles and the subsequent translation to clinical use.

## Introduction

Human hair follicle (HF) development occurs during embryogenesis in the second trimester of gestation and relies on coordinated signaling of morphogens in the neuroectodermal-mesodermal regions. This process involves a complicated, gradient-dependent interplay, with a variety of participating biochemical pathways such as wingless and Int-1 (WNT)/β-catenin, ectodysplasin A (EDA), Sonic hedgehog (SHH), Notch, and bone morphogenetic proteins (BMPs), that result in changes in the fate of HF progenitor cells present in both cell layers of the skin. These processes result in the differentiation of the different components of HFs (Christiano, 2004). This HF differentiation can be divided into three phases: induction, organogenesis, and cytodifferentiation (Rishikaysh et al., 2014).

Stem cell-based tissue engineering aiming to reconstruct HFs *in vitro* to replace lost or damaged HFs due to disease, injury, or aging to eventually restore hair growth is gaining increasing attention. Although much progress has been made, clinical applications of cell-based therapies for hair loss have not been developed. This is most probably due to the knowledge gap regarding the precise mechanisms of human HF organogenesis during embryonic development, and replication of the process *in vitro*. The lack of proper studies on human embryonic material hampers research on *in vitro* HF neogenesis and precise knowledge of the early processes of HF organogenesis. Moreover, most studies on HF organogenesis have focused on the biomolecular pathways involved and not on achieving HF neogenesis *in vitro*. In regenerative biology, it is generally acknowledged that matrix molecules, and their physicochemical characteristics, are involved in the control and completion of organogenesis, but this has hardly been explored in the context of HF morphogenesis (de Groot et al., 2020). It is known that signaling pathways required for early HF morphogenesis are evolutionarily conserved between species (Botchkarev et al., 2003; Wu et al., 2004). Nevertheless, significant differences exist in the expression of molecular signals between different species.

To help move research on human HF neogenesis forward, this review aims to collate information on HF development from the perspective of cell-based therapy. We will not only describe the morphological stages, and the relevant cellular and molecular processes in the developing human fetus, but also the influence of the extracellular matrix (ECM) on HF fate. Due to the limited availability of human data, we could not restrict our review to human research only, and we had to include research from other species when discussing HF induction, organogenesis, and cytodifferentiation. We then discuss how current knowledge of early HF development in the human fetus can be implemented in novel techniques to replicate cellular and molecular signaling for *ex vivo* HF neogenesis.

## Cellular Changes During HF Development in the Fetus

### Induction of the HF

**FIGURE 1:**
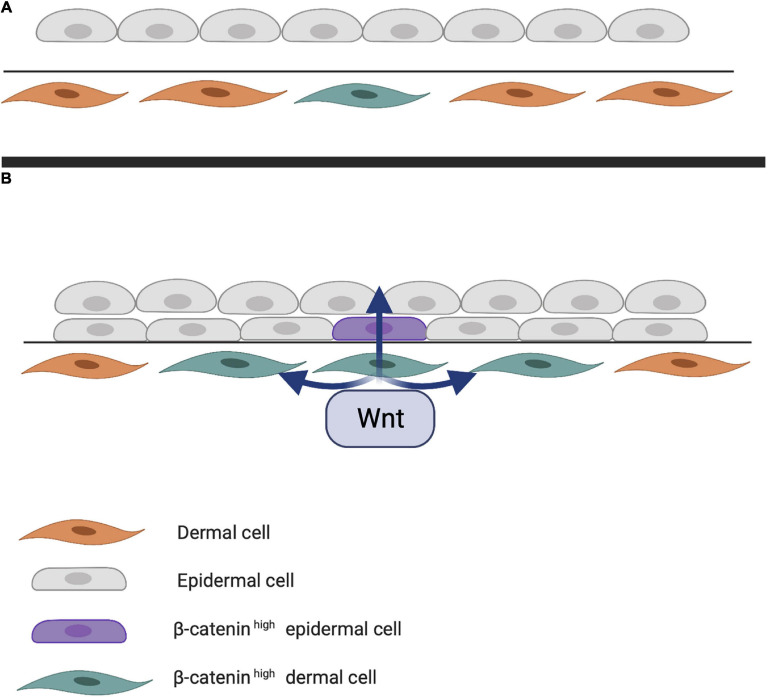
**(A)** First step of hair follicle (HF) induction. Through an unknown trigger, intracellular β-catenin accumulates in the dermis, activating various transcription factors in the nucleus. **(B)** Next step of HF induction. Intracellular dermal β-catenin induces expression of WNT ligands that activate neighboring cells and eventually those in the epithelial layer. Epithelial WNT activation results in a high concentration of intracellular β-catenin in the epidermis.

**FIGURE 2:**
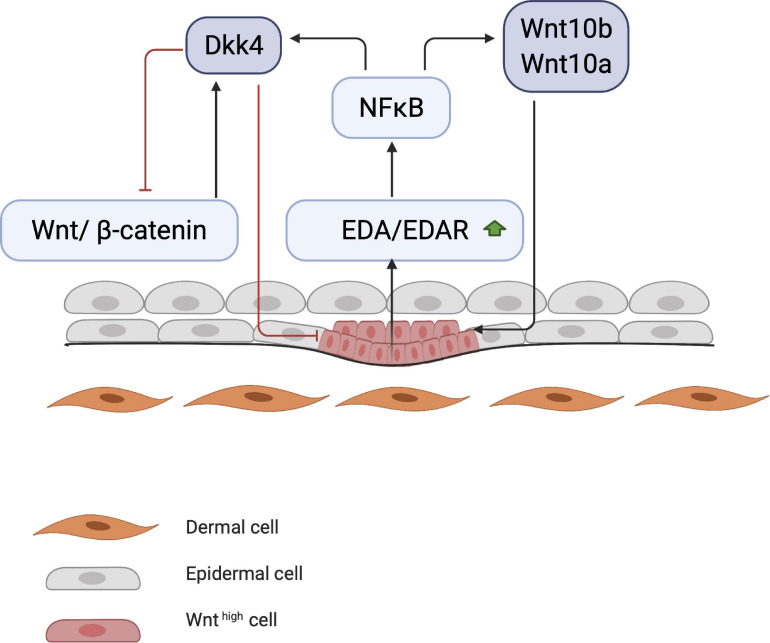
Start of placode formation. During placode formation, NF-κB produces inhibitory signals (DKK4) and stimulates WNT molecules (WNT10a and WNT10b). Stimulating and inhibitory signals create a WNT concentration gradient, where WNT^high^ concentration results in placode formation.

Early studies on chick embryos showed that the first signal leading to appendage development most likely comes from dermal cells through activation of the WNT pathway due to accumulation of subcellular β-catenin (Noramly et al., 1999). β-catenin in dermal cells appears to be the first trigger for inducing HF placode formation in the epidermis, but the underlying mechanisms of β-catenin accumulation remains unknown (Figure 1). At first, β-catenin is only present in the cytoplasm of a small number of dermal cells (perhaps even in a single cell), after which it accumulates in the cytoplasm, and is translocated to the nucleus, where it binds to members of the TCF/LEF family of transcription factors, inducing WNT ligand expression. Eventually, the surrounding dermal cells express WNT ligands, followed by epithelial cells (Huelsken et al., 2001). In addition to WNT/β-catenin, the EDA/EDA receptor (EDAR) pathway likely plays one of the earliest roles in HF development (Schmidt-Ullrich and Paus, 2005; Fuchs, 2007). These pathways are essential for NF-κB activation, and EDA/EDAR/NF-κB signaling is required to refine the pattern of WNT/β-catenin activity (Zhang et al., 2009). The expression of EDA and EDAR is positively regulated by WNT/β-catenin pathway, with WNT10b and WNT10a appearing to be the main activators (Figure 2; Durmowicz et al., 2002). Similar to β-catenin, WNT10b is a direct target of the EDA/EDAR pathway, and the NF-κB signal is essential for sustaining high levels of WNT10b and WNT10a (Zhang et al., 2009). It is believed that in the epidermis during placode formation, EDAR signaling levels are controlled by the inhibitory WNT signaling molecule Dickkopf-related protein 4 (DKK4), which results in a negative feedback loop of expression of the WNT proteins. In addition, expression of DKK4 is regulated by NF-κB as well as LEF/TCF/β-catenin (Zhang et al., 2009; Cui et al., 2010).

Thus, with regard to *Eda*/*Edar* gene expression, WNT signaling possesses both stimulatory as well as inhibitory functions (Mou et al., 2006; Zhang et al., 2009). A controlled gradient between these placode-promoting and inhibitory morphogens is required to define the borders of the placode in order to establish a regular array of placodes (Sick et al., 2006). It is believed that these secreted factors compete with each other in a delicately balanced reaction-diffusion system. Compared with the smaller WNT inhibitors such as DKK1 and DKK4, the larger hydrophobic WNT activators tend to remain around the placode for a longer period (Zhang et al., 2009). In transgenic mice, during placode formation, the WNT inhibitor DKK1 is overexpressed in the epithelium, blocking WNT signaling in both the epithelial and mesenchymal layers. This results in a complete attenuation of the formation and downgrowth of the placode (Andl et al., 2002). In contrast, overexpression of DKK4 (in the epidermis) has seemingly no effect on the formation of the placode.

**FIGURE 3:**
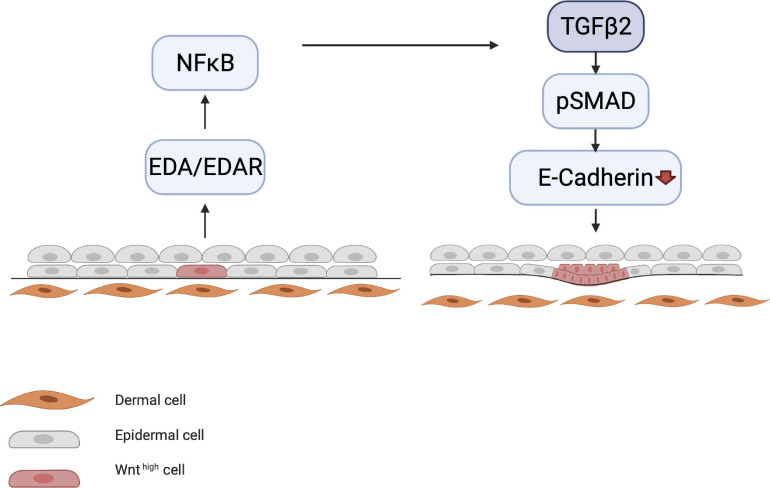
Downregulation of E-cadherin. EDA/EDAR signaling is downstream of epithelial WNT activation. EDA/EDAR signaling activates NF-κB, which in turn leads to the production of TGF-β2 by an as yet unknown mechanism. During the induction phase, TGF-β2 downregulates E-cadherin via the pSMAD pathway. E-cadherin downregulation leads to the migration of epidermal cells, which is a prerequisite for placode formation.

Meanwhile, other processes occur simultaneously, such as the initiation of motility of the epithelial cells by E-cadherin downregulation, which is governed by the NF-κB/LIM homeobox 2 (LHX2)/transforming growth factor (TGF)-β2 axis. LHX2 activates TGF-β2, which downregulates E-cadherin via phosphorylation of focal adhesion kinase (Tomann et al., 2016). E-cadherin is involved in cell–cell contact; its downregulation causes cells to become motile, which is essential for placode formation and invagination into the dermis (Figure 3).

### Organogenesis of the HF

**FIGURE 4:**
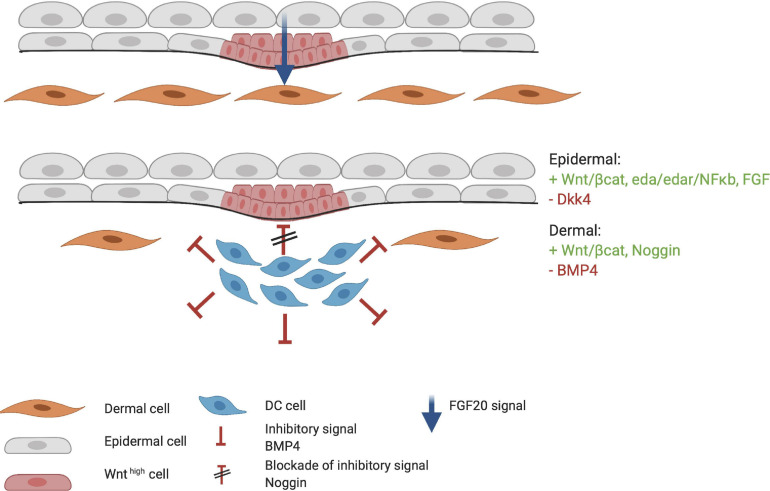
Formation of the dermal condensate (DC). FGF20 is the first epidermal signal that marks the start of the organogenesis phase. FGF20 induces DC formation in the dermis. DC cells produce inhibitory factors such as BMP4 to fine tune the borders and specification of the DC. At the apical side of the DC, high concentrations of Noggin block the inhibitory signaling of BMP4. Meanwhile, the placode continues to mature through the FGF, WNT/β-catenin, and EDA/EDAR/NF-κB axis. Red indicates inhibitory signaling, and green indicates stimulatory signaling.

During HF organogenesis, epithelial cells of the placode interact with surrounding dermal cells. Subsequently, the dermal cells proliferate and form a pre-dermal condensate (DC). In turn, signaling molecules from the pre-DC interact with epithelial cells and induce their invasion into the dermis (Abe and Tanaka, 2017). DC formation marks the start of the next phase of HF organogenesis. The production of fibroblast growth factor 20 (FGF20) is the first epidermal signal required for the formation and maintenance of a DC during the subsequent stages (Figure 4; Ahtiainen et al., 2014; Biggs et al., 2018; Saxena et al., 2019). The WNT/β-catenin and EDA/EDAR/NF-κB signaling pathways may also contribute to the formation of the pre-DC; however, the significance of their involvement remains unknown. WNT likely supports FGF20 epidermal signaling to support the DC (Tsai et al., 2014). Indeed, the WNT10a and WNT10b ligands are upregulated in the placode when DC organogenesis begins (Gupta et al., 2019). As DC formation progresses, the BMP concentration around it increases and inhibits HF induction of neighboring epidermal cells (Sennett and Rendl, 2012). BMP4 likely reinforces the lateral inhibition propagated by DKK4 signaling, which is also involved in placode formation (Huh et al., 2013). This inhibitory signaling acts in concert with the stimulatory WNT pathway (Figure 4). The BMP inhibitor Noggin (NOG) is expressed in the DC and is thought to act as a short-range BMP inhibitory signal that promotes placode boundary formation. When NOG is overexpressed, placode formation lasts longer and is disproportional in size, whereas in NOG-deficient mice, organogenesis is interrupted (Plikus et al., 2004). Furthermore, in transgenic mice carrying a WNT-responsive *Lef1* reporter gene, high levels of LEF1 are expressed in the DC; however, in the absence of epithelial WNT/β-catenin signaling, the DC fails to develop (Van Mater et al., 2003). Thus, WNT is considered essential throughout organogenesis. During DC formation, dermal fibroblasts that undergo transition toward a DC phenotype, can be distinguished from other cells by the high expression levels of the stem cell marker SOX2. On the other hand, compared with fibroblasts, the cells in the DC highly express FOXD1, another stem cell marker (Fetting et al., 2014; Mok et al., 2019). When the DC matures, due to differential cellular changes, cells polarize around the basal-apical axis and SOX2 and FOXD1 expression increases. Expression of the fibroblast characteristic protein TWIST2 is lost as DC maturation becomes more prominent (Mok et al., 2019).

**FIGURE 5:**
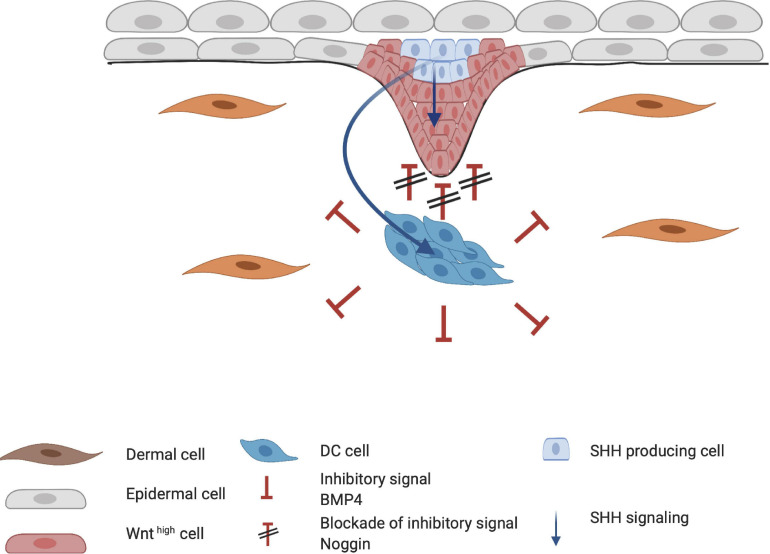
Influence of Sonic hedgehog (SHH) on placode formation. SHH affects both the epithelial and mesenchymal layer of the developing hair follicle. SHH aids in the proliferation of the hair placode in the epithelial layer through cyclin D1. In the dermal condensate (DC), SHH primarily induces higher Noggin expression, creating a positive feedback loop for the downgrowth of the hair placode.

When HF placode and DC formation are completed, a second epidermal signal is introduced from the placode. SHH and platelet-derived growth factor (PDGF) signaling pathways are involved in the secondary wave that initiates downgrowth of the placode to form the hair germ (Figure 5; St-Jacques et al., 1998; Karlsson et al., 1999). SHH expression is regulated by the placodal EDAR/NF-κB pathway and is crucial for the progression of organogenesis. Early studies with *Shh*-knockout mice showed that even when WNT10b, BMP4, and LEF1 are expressed (required for normal induction of the placode and DC), the hair germ did not form (St-Jacques et al., 1998). This suggests that SHH signaling occurs downstream of these inductive molecules. Accordingly, both *Wnt*- and *Eda*-null mice do not express SHH, indicating that WNT and EDA are required for SHH signaling (Pummila et al., 2007). The initial role of epithelial SHH during organogenesis consists of stimulating keratinocyte proliferation in the placode, indirectly aiding formation of the hair germ (Abe and Tanaka, 2017). Moreover, in the placode, cyclin D1 induces keratinocyte proliferation toward hair germ formation (Figure 5; Abe and Tanaka, 2017). D-type cyclins typically drive cell cycle progression through the G1 phase and both SHH and WNT10b signaling can induce cyclin D1 expression (Rishikaysh et al., 2014). This demonstrates the important role of SHH in cyclin D expression (Andl et al., 2002; Schmidt-Ullrich and Paus, 2005).

**FIGURE 6:**
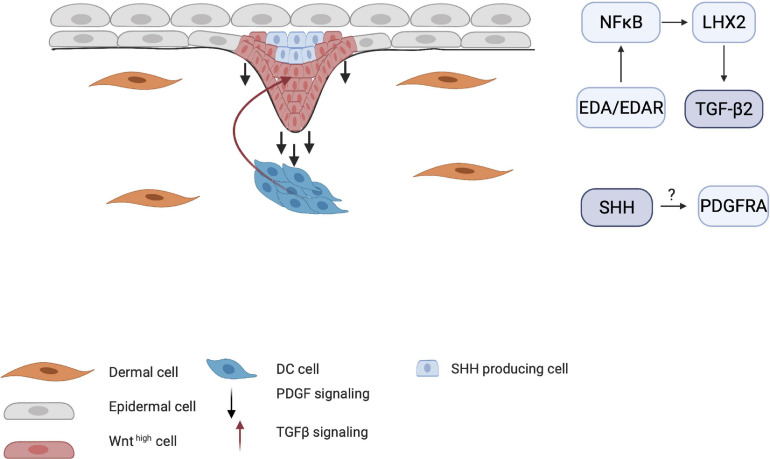
Influence of TGF-β and PDGF on placode formation. Although the mechanisms by which PDGF receptors are induced are unknown, PDGFRA-positive cells become more restricted around the DC as maturation continues. TGF-β2 is also crucial for HF downgrowth through the NF-κB/LHX2 pathway through establishment of a balance between stimulating and inhibitory WNT concentration gradient.

The importance of PDGF for HF development became clear when *Pdgfa*-knockout mice demonstrated abnormal hair development (Karlsson et al., 1999). The interplay between SHH and PDGF receptor A (PDGFRA) has also been demonstrated by Karlsson et al. (1999), who found that early follicular mesenchymal aggregates, or the pre-papillae, consist of densely packed PDGFRA-positive cells. These clusters failed to form in the skin of *Shh*-knockout mice, indicating that SHH mediates the aggregation of PDGFRA-positive dermal cells into dermal pre-papillae. Under normal conditions, PDGFRA expression becomes more concentrated in the dermal pre-papillae areas and progressively becomes restricted to the epithelial hair placode as the DC matures into the dermal papillae (Figure 6). This PDGF signaling pathway is a clear example of an interaction between the dermis and epidermis, as PDGFRA is uniquely expressed in the dermis. *Pdgfra*-knockout mice that survive show normally developed HFs, indicating that the PDGF pathway is not essential in HF organogenesis.

Transforming growth factor-β has also been implicated in HF organogenesis. Indeed, *Tgf*-β receptor-knockout mice show underdeveloped HFs (Qiu et al., 2011). Recent studies have shown that TGF-β signaling in late organogenesis during HF downgrowth occurs via the EDA/EDAR/NF-κB pathway through the LHX2 axis (Tomann et al., 2016). Mice that lack LHX2 do not show TGF-β expression and have reduced numbers of HFs. This indicates that the EDA pathway is crucial in both the induction and organogenesis phases of HFs.

### Cytodifferentiation of the HF

During the last stage of development, cells differentiate into all components of the HF, including the inner root sheet (IRS), outer root sheet (ORS), and hair shaft. The differentiation of all components is regulated by multiple signaling molecules, such as GATA3, CCAAT displacement protein (CDP), and BMPs for IRS; SOX9 and SHH for ORS formation; and WNT/β-catenin, Notch, BMPs, and FOXN1 for the expression of selected hair shaft keratins and/or hair shaft development (Schneider et al., 2009). Mutations in GATA3, a zinc finger transcription factor, and CDP, a regulator of differentiation-specific gene expression, leads to a complete failure of IRS formation (Kaufman et al., 2003). When the BMP receptor *Bmpr1a* is knocked out in mice, highly proliferative epidermal placode cells remain undifferentiated and also fail to form the IRS (Ming Kwan et al., 2004). BMP and Notch pathways suppress epithelial growth, and loss of these factors or their receptors can eventually lead to tumor formation in human HFs (Kan et al., 2011).

The Notch family consists of highly conserved transmembrane receptors that regulate cell fate through cell-cell and cell-ECM contacts. For instance, the cell fate of keratinocytes in the hair placode is regulated by Notch via modulation of the keratinocyte adhesion properties (Watt et al., 2008). Interestingly, in *Notch1*-null mice, the number of placodes is not altered compared with the wild type. This indicates that the Notch pathway is not essential for the HF induction and organogenesis phases (Vauclair et al., 2005). RBP-J, or the recombination signal binding protein for the immunoglobulin kappa J region, is a transcriptional regulator that is important in the Notch signaling pathway. The mice that lack the *Rbp-j* gene in keratin 14-expressing cells have smaller follicles when compared to control mice, and show no further maturation of the HFs after birth. The *Rbp-j*-knock-out mice also express lower levels of the IRS marker GATA3. This suggests that Notch/RBP-J is required in the early stages of IRS cytodifferentiation (Blanpain et al., 2006).

Bone morphogenetic proteins also appear to play a central role in Notch1 expression. FOXN1 is required for the expression of Notch1, which is also dependent on BMP2 and BMP4 (Cai et al., 2009). Moreover, GATA3 is involved in downregulating Notch1 in the developing hair matrix, which is controlled by BMPs through FOXN1 (Kobielak et al., 2003). Studies by Estrach et al. (2006) found that regions in the developing HF that eventually become the hair shaft highly express both WNT/β-catenin and Notch, suggesting that these pathways are closely linked. If either of the pathways is blocked during the cytodifferentiation phase, the HFs are converted into cysts. They also found that Notch is a downstream target of β-catenin for determining epidermal cell fate in the HF; when the Notch ligand JAG1 is knocked out in mice, hair regrowth is inhibited (Estrach et al., 2006).

## Impact of the ECM on HF Development

Hair follicle development involves a complex interplay of reciprocal mesenchymal and epithelial signaling pathways. The fate of most cells is controlled by an interaction of extrinsic signals with a cascade of intrinsic transcriptional pathways. It is known that a great amount of external signaling is provided by the microenvironment of the cell (Sennett and Rendl, 2012). Indeed, the significance of the ECM during skin development has been demonstrated in many studies (Raghavan et al., 2000; Chen et al., 2015; Morgner et al., 2015). There is also growing evidence that the ECM is important for HF progenitor cell fate determination throughout HF development (Chermnykh et al., 2018).

In the skin, the basement membrane (BM) connects the epidermis to the dermis. The BM is important in HF development because of the unique molecular and structural composition of laminins and collagens (Brakebusch et al., 2000). Laminins in the BM are linked to transmembrane receptors-primarily β4 integrins-in the epithelium. β5 integrins are mainly linked to collagens, while β1 integrins can bind both laminins and collagens (Hynes, 1992). Collectively, these proteins and adhesion complexes in the BM are essential for the structural and functional integrity of the skin. Mutations in BM-associated proteins can lead to various skin diseases and aberrant appendages (Breitkreutz et al., 2013).

Laminins are trimeric proteins that consist of α, β, and γ polypeptides. In mammals, there are five distinct α, three β, and three γ laminin chains, which may yield many different laminin trimers. To date, 16 laminins have been discovered (Domogatskaya et al., 2012; Hohenester and Yurchenco, 2013). Laminins are essential in the BM, and because the BM is involved in multiple structures of the skin, including adipocytes, blood vessels, and nerves, multiple laminins are found in the skin (Aumailley and Smyth, 1998). Here, we will focus on the epidermal BM, which mainly consists of laminin (α3β3γ2)332, laminin (α3β1γ1)311, and laminin (α5β1γ1)511, which are all expressed by keratinocytes.

Collagens provide tensile strength to tissues, and 20 of the 28 collagens can be found in the skin (Ricard-Blum, 2011). The main types of collagen in the skin are type I, II, III, and IV, where type I is the most abundant (Lodish et al., 2008). Here, we will focus on the collagens present in the BM. In addition to collagen IV, which is abundantly present in the BM of adult human skin, type VII collagen is ubiquitously present in the developing fetal BM and is involved in HF development (Karelina et al., 2000).

The integrin family of transmembrane proteins consists of heterodimeric α and β subunits that form a bridge between the ECM and cells (Hynes, 1992). Integrins α2β1, α3β1, and α6β4 are the most prominent integrins found in the skin, and are secreted by basal keratinocytes that are attached to the BM (Watt and Hertle, 1994). Integrins are involved in various processes such as cell migration and adhesion, proliferation, and differentiation as well as apoptosis (Brakebusch et al., 1997), and can translate mechanical signals from the external environment into intracellular responses (Tsimbouri et al., 2014).

In the following sections, we will further discuss the involvement of laminins and collagens–the two major fibrous proteins in the skin–in HF development, as well as integrins, which are cellular transmembrane proteins that act as mechanotransducers within the ECM.

### Importance of the ECM for HF Induction

**FIGURE 7:**
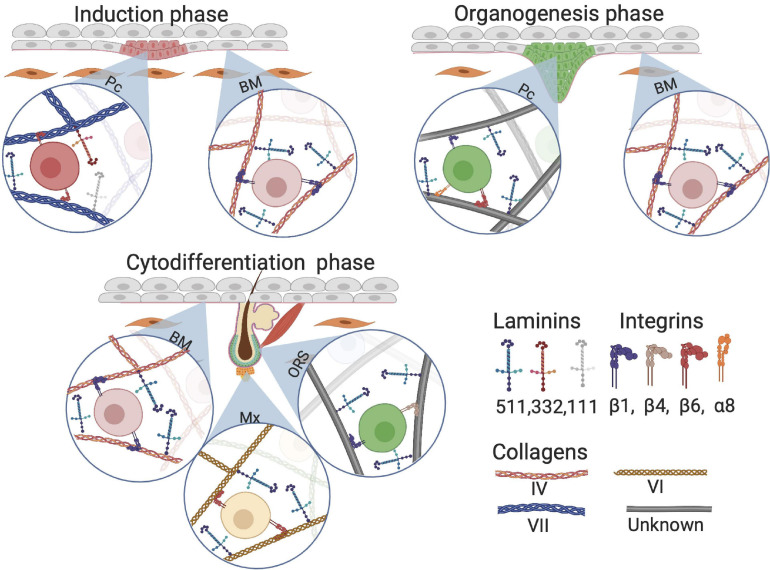
Composition of extracellular matrix (ECM) proteins at different phases of HF development. Although Collagen VII anchors on collagen I fiber, we visualized collagen VII as a classical collagen fiber for simplicity. BM, basement membrane; Pc, hair follicle placode; Mx, hair matrix; ORS, outer root sheet.

In early HF development, laminin 511 is important as it is one of the first upregulated laminins when elongating hair germs emerge (Li et al., 2003). *Lama5*-knockout mice, lacking laminin 511, exhibit fewer hair germs compared with the control group. Additionally, expression of the early HF markers SHH and Gli1 is lower in *Lama5*-knockout mice than that in control, implicating involvement of laminin 511 in early HF morphogenesis (Li et al., 2003). Gli1 is required for fetal HF progenitor cells to eventually drive HFs toward the anagen phase and is a downstream target of SHH (St-Jacques et al., 1998). These HF precursor cells eventually migrate toward a particular region, the future HF bulge (Abe and Tanaka, 2017). Interestingly, treatment of *Lama5*−/− skin with exogenous laminin 511 fully reversed the BM defects and restored HF development (Li et al., 2003). During the early HF-inductive phase in mouse, laminin 332 is briefly upregulated but does not appear to have a crucial role in further HF development when compared with laminin 511 (Nanba et al., 2000). Furthermore, laminin 511 is universally expressed in the BM in contrast with the locally expressed laminin 332, which may illustrate their complementary roles in HF neogenesis: the ubiquitous presence of laminin 511 is required for the entirety of HF neogenesis, whereas laminin 332 is solely required during the inductive phase (Figure 7).

Research on collagens in HF development is limited. Nevertheless, one study found that during human skin morphogenesis, protein and mRNA levels of collagen VII increase with increasing gastrulation age (Karelina et al., 2000). At the start of HF morphogenesis around gestational age 13–15 weeks, there is a loss of type VII collagen in the BM around the HF placodes. This supports the hypothesis that ECM is involved in HF development and downgrowth of the placode, when HF induction progresses (Figure 7; Karelina et al., 2000). Interestingly, no changes were found in collagen IV distribution in the BM during the induction phase of HF development (Karelina et al., 2000).

Integrins have been shown to play a crucial role in embryonic skin development, including induction of the HF placode. Mice with β*1 integrin* subunit-knockout in keratin 14-positive cells exhibit very thin and fragile skin without HFs. After birth, mice pups die due to a lack of proliferative epidermis and dehydration (Raghavan et al., 2000). Raghavan et al. (2000) also found that of all integrins, β1 integrin had the most dramatic effect on BM formation. After conditional ablation of β1 integrin, the epidermis cannot attach to the ECM and HFs fail to develop due to defects that arise during invagination of the epidermis into the underlying dermis. β1 integrin primarily plays a role in BM formation, and the attachment of the dermis to the epidermis; therefore, the role of β1 integrin in HF formation is indirect. In contrast, α6 integrin appears to play an important role in HF development, as decreased expression of α6 integrin leads to a delay or complete cessation of skin appendage development (Rippa et al., 2013).

### Role of the ECM in HF Organogenesis

Studies on chick embryos showed that dermal fibroblasts that eventually become DC cells are dependent on β1 integrin for migration (Michon et al., 2007). Furthermore, the maturation of cells from pre-DC into DC is dependent on a Notch/integrin interaction (Michon et al., 2007). Additionally, mesenchymal cells associated to the HF bulge express the β1 integrin subtype α8β1, which is linked with the arrector pili muscle. HFs in *Integrin* α*8*−/− skin fail to anchor the arrector pili muscle to the HF bulge (Fujiwara et al., 2011). Epithelial-derived laminin 511 interacts with mesenchymal β1 integrin, promoting the formation of primary cilia–small microtubule-based appendages that are present on most cells in the body. Primary cilia are widely present in DCs and mediate the initiation of epithelial-derived SHH signaling through activation of downstream SHH effectors (Gao et al., 2008). Inhibition of the laminin 511 receptor, β1 integrin, halts primary cilia formation and inhibits further development of HFs (Gao et al., 2008). However, in a later publication of DeRouen et al. (2010) laminin 511 seems to promote epithelial downgrowth without affecting primary cilia. Moreover, laminin 511 triggers NOG expression, which is PDGF- and SHH-dependent; this indicates that laminin 511 is important for DC maintenance (Figure 7). As HF development proceeds, the composition of laminin changes; laminin 511 expression shows almost no change, whereas laminin 322 and laminin 111 are downregulated (Fleger-Weckmann et al., 2016). Unfortunately, the role of collagens during HF development is not known yet.

### Influence of the ECM on HF Cytodifferentiation

During HF morphogenesis, laminins have been implicated in the regulation of HF development via crosstalk between the mesenchyme and epithelium (Schmidt-Ullrich and Paus, 2005). The γ1 component of laminins, which is encoded by *LAMC1*, is the most abundant in the BM (Durbeej, 2010). When *Lamc1* is knocked out in mice, embryonic development is halted at day 5.5 (Smyth et al., 1999). However, mice lacking *Lamc1* in basal keratinocytes only show the loss of laminin 511 and 211 (Fleger-Weckmann et al., 2016), as well as reduced hair matrix cells, and reduced differentiation into hair shafts; nevertheless, placode numbers and hair germ formation are unaffected in these knockout mice. The effect of *Lamc1* on laminin 311 remains unknown because the presence of laminin 311 in murine skin is controversial (Fleger-Weckmann et al., 2016).

The collagen that is most widely expressed in the HF is collagen VI, which is also known to contribute to wound healing (Figure 7). *Col6a1*-knockout mice show a decreased HF phase length in adulthood (Chen et al., 2015). In addition, collagen VI may be involved in the WNT/β-catenin pathway during fetal and adult HF development (Iyengar et al., 2005; Chen et al., 2015). Although collagen IV is the most abundant collagen in the BM, its expression remains unchanged throughout all the stages of HF development (Alfayez, 2010). In the late anagen phase in adult skin, a continuous expression pattern of collagen IV is present along the whole HF, while other proteins such as integrin α6β4, laminin 511, and collagen VII show weak or negative expression at the basal part of the follicle (Chuang et al., 2003). This suggests that collagen IV is not required for HF morphogenesis or other stages in the development of the hair (Alfayez, 2010).

The importance of β1 integrins has been demonstrated in *Integrin* β*1*-knockout mice, in which different phenotypic changes were observed, including epidermal thickening, defective BM, and malfunctioning HFs (Brakebusch et al., 2000). It has also been shown that β1 integrins are important for the proliferation of HFs during cytodifferentiation and the adult anagen phase, but are not necessary for the induction of HFs. Moreover, Brakebusch et al. (2000) found that β1 plays an essential role in the maintenance of the BM (Figure 7).

## Tissue Engineering

Techniques in regenerative medicine for regrowing HFs have recently gained increasing attention. To achieve regeneration, HF-derived epithelial and mesenchymal HF progenitor cells need to be differentiated through an orchestrated interplay between individual cells and morphogens in conjunction with their ECM. Recently, organoids were successfully established from human-induced pluripotent stem cells, which eventually generated HFs (Lee et al., 2020); similar to early development, the researchers supplemented pluripotent stem cells with the appropriate ECM and morphogens such as BMP4, FGF, and TGF-β inhibitors. Although these findings seem promising, restoring hair growth in adult humans remains very challenging. This is mostly because the pathology behind most forms of hair loss is not fully understood at the cellular level. Studies that succeeded in replicating hair growth *ex vivo* did not show the desired effect of generating fully functional HFs, possibly due to the absence of physiological conditions.

In the following sections, we will outline three key elements for hair regeneration–HF progenitor cells that reside in HFs, morphogens that are known to play a role in fetal development, and the ECM–and discuss the required combined strategies for achieving HF regeneration as well as neogenesis.

### HF Progenitor Cell Sources for Bioengineering HFs

The most rational way of achieving HF neogenesis *in vitro* is by combining inductive mesenchymal and receptive epithelial HF progenitor cells from a healthy scalp donor area to mimic the induction phase of fetal skin at gestation week 15. In the fetus, this leads to a well-orchestrated interaction that repeats itself in adult HF cycle lifelong. Unfortunately, these interactions between mesenchymal and epithelial HF progenitor cells are negatively impacted during alopecia (Garza et al., 2011). Recent studies have shown that scalp-derived HFs from patients with androgenic alopecia have normal HF bulge cell populations, but their dermal papilla (DP) cell source shows altered gene expression compared with that of healthy patients (Garza et al., 2011; Chew et al., 2016).

A possibility to replace the diseased DP is to use DP from a healthy donor, or an autologous non-balding area. Two decades ago, Reynolds et al. (1999) demonstrated that the HF end bulb is capable of allogenic regrowth of HFs without initiating an immune reaction and the eventual rejection of the transplanted end bulb due to the lack of major histocompatibility complex 1 (MHC1). Thus, based on the low immune response of allogenic HFs, it is possible to receive HFs from non-alopecia donors. However, recent studies have shown that allogenic HF transplantation is only possible alongside life-long immunosuppression (Kim et al., 2018). These discrepant immune responses may have resulted from the use of the HF end bulb by Reynolds et al. (1999) and the whole HF by Kim et al. (2018).

Therefore, although allogenic micro-dissected DP and dermal sheath transplantation have been proven to induce HFs, the best approach for HF regeneration includes using both autologous mesenchymal and epithelial cell sources from healthy areas of skin biopsies and/or scalp HF grafts, because immunosuppressants can have severe side effects.

#### Importance of the DP

Due to ethical regulations concerning the use of human material, few studies have used human DP cells for *in vivo* HF regeneration in humans. One study reported that a heterologous transplantation of end bulb HFs (including DP) was able to survive and form a HF (Jahoda et al., 1984). However, most DP studies are based on animal experiments in rodents. Early *in vivo* studies in rodents showed that isolated DP structures are capable of (re)generating HFs when implanted in the recipient area (Jahoda et al., 1984; Horne et al., 1986). These studies showed that DP structures, unlike dermal fibroblasts, are capable of differentiating epidermal keratinocytes into a follicular phenotype. More recent studies have shown that the DP is necessary for initiating HF regeneration in rodents (Rompolas et al., 2012). However, human HFs can only be induced in the skin of nude mice when human DP cells are mixed with a human epidermal cell source (Abaci et al., 2018). This indicates that both mesenchymal and epithelial cell sources are needed to achieve human HF neogenesis.

#### DP Cells *in vitro*

Since the DP is crucial for HF neogenesis, substantial effort has been devoted to expanding DP cells *in vitro*. The commonly used technique for this is surgical microdissection of human DP followed by 2D culture to allow cellular outgrowth of the tissue. Instead of outgrowth from the DP, cells can also be isolated by enzymatic digestion prior to 2D culture (Wu et al., 2005; Topouzi et al., 2017). However, when DP cells are cultured in 2D culture systems, they rapidly lose their inductive capacity, and signature genes such as alkaline phosphatase (*ALPL*), *NOG*, *WNT inhibitory factor 1* (*WIF1*), and *Versican* (*VCAN*) are significantly suppressed (Ohyama et al., 2012). This drawback can be avoided by changing the culture conditions to a 3D culture, after 2D expansion at a low passage number. During 3D culture, DP cells form spheres, re-introducing cell–cell contacts, and restoring the DP transcriptional signature (Higgins et al., 2010). DP cells can be distinguished from dermal fibroblasts according to their aggregative behavior, as they form spheres when placed in 3D culture. Moreover, after DP sphere formation, DP signature genes such as *ACTA2*, *ALPL*, and *VCAN* are upregulated (Osada et al., 2007).

#### Epidermal HF Progenitor Cells *in vitro*

Both DP and epidermal cells alter their properties when cultured *in vitro*; however, HF induction is possible in 3D culture when the ECM is cocultured with these two cell types. Under such conditions, the ECM and morphogen gradients can be controlled to mimic the natural (fetal) conditions. In addition, it has been reported that CK15+-HF bulge cells are prominently involved in HF neogenesis and repair *in vivo* (Ito et al., 2005; Al-Refu et al., 2009). Ablation of CK15+ cells in the bulge area leads to complete loss of HFs *in vivo*, indicating that this specialized epithelial cell source, as well as DP cells, is required for HF organogenesis and maintenance (Ito et al., 2005). Thus, CK15+-HF bulge cells as well as DP cells should be supplemented in *in vitro* systems.

#### *In vitro* and *in vivo* Bioengineered Germs Using Different Cell Populations

**FIGURE 8:**
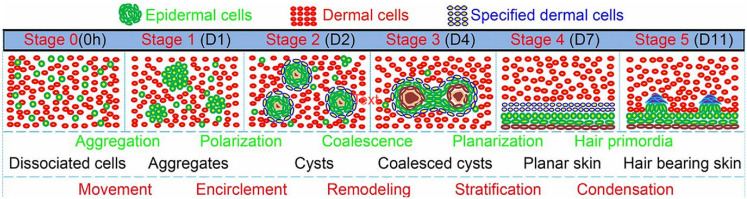
Morphological transition of the cells into six different phases: dissociated cells, cell aggregates, polarized cysts, cyst coalescence, planar skin, and hair-bearing skin. The first four stages are *in vitro* and the last 2 are *in vivo*. Adapted from Lei et al. (2017).

The most straightforward strategy for inducing (re)generation of the HF is thought to be the combination of both DP and HF bulge cells to effectively mimic the start of anagen phase *in vivo* (Roh et al., 2004). Multiple studies have shown that combining DP cells with an epithelial component such as HF bulge cells has a positive effect on the engineering of hair germs toward hair specific differentiation *in vitro* (Roh et al., 2004; Toyoshima et al., 2012; Kalabusheva et al., 2017; Nilforoushzadeh et al., 2017; Gupta et al., 2019). 3D time-lapse imaging of bioengineered HF germs, consisting of fibroblast and keratinocytes from the skin of newborn mice mixed in a collagen I gel, showed morphological transition of the cells into six different phases: dissociated cells, cell aggregates, polarized cysts, cyst coalescence, planar skin, and hair-bearing skin (Figure 8; Lei et al., 2017). The first four stages were achieved *in vitro*, while the last stages were observed when the germs were transplanted into nude mice (Lei et al., 2017).

Bioengineered HF germs containing both rodent DP and keratinocytes recapitulate HF structures comparable to native HFs after transplantation in the skin of nude mice (Tezuka et al., 2016). To date, most researchers still use *in vivo* mouse models to track HF neogenesis, mainly due to vascularization issues when generating HF-bearing skin models *in vitro*. Vascularization of an *in vitro* model is essential, because the supply of sufficient oxygen and nutrients is required to support the neogenesis of complex structures, such as HFs, in the skin.

**FIGURE 9:**
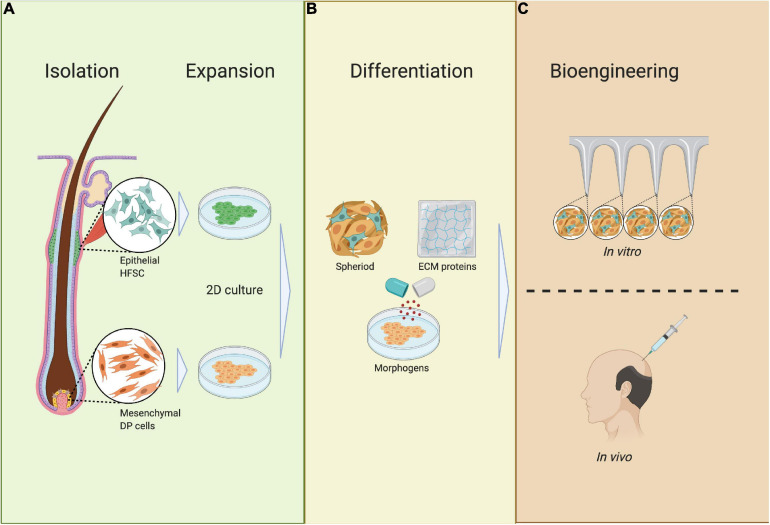
Requirements of bioengineered HF germs. **(A)** Isolation and expansion; epithelial HF stem cells (HFSCs) and mesenchymal dermal papilla cells (DPCs) are isolated from the HF during the anagen phase, after which HFSCs and DPCs are expanded in 2D culture. **(B)** Differentiation; HFSCs and DPCs are mixed together with ECM proteins and differentiated into spheres in 3D culture. **(C)** Bioengineering; HFSC- and DP-containing spheres are placed in an ECM scaffold or full skin equivalent to track HF neogenesis or are transplanted *in vivo*.

While vascularized skin models appear promising for HF neogenesis in rodents, *in vivo* reconstruction of HFs with human cells has not been successful. However, Ehama et al. (2007) managed to induce HF neogenesis from a chimeric mixture of murine DP cells and human epithelial cells. Another study demonstrated human HF neogenesis *in vitro* (Lindner et al., 2011); DP cells, epithelial cells, and melanocytes were isolated via microdissection and expanded *in vitro* in 2D culture (Figure 9). DP cells were then grown into spheres called neopapillae and coated with ECM proteins of the BM, such as collagen IV. Thereafter, melanocytes and epidermal cells mixed with the neopapillae to eventually form hair-containing microfollicles with all relevant cell types and structures (Figure 9; Lindner et al., 2011). Additionally, a recent study with neopapillae incorporated in a human full skin equivalent showed an epithelial downgrowth of keratinocytes toward DP spheres and differentiation toward an IRS precursor (Vahav et al., 2020).

### The Influence of Morphogens on Bioengineered Human HFs

Hair follicle neogenesis from bioengineered hair germs involves complex interactions between mesenchymal DP cells and epithelial HF progenitor cells. To construct bioengineered hair germs, the epithelial–mesenchymal interaction must be guided by a microenvironment that mimics the *in vivo* conditions as closely as possible. This can be partly achieved by biomolecular signals derived from DP cells. However, DP cells quickly lose their inductive capacity *in vitro*, and thus, additional growth factors have to be supplemented when bioengineering hair germs (Figure 9; Ohyama et al., 2012).

Two very potent morphogens involved in both the human adult anagen phase, and the organogenesis phase for HF development are FGF20 and PDGF. Studies have shown that when DP cells are supplemented with FGF2 they become more proliferative and their inductive capacity is restored (Lin et al., 2016). Dermal cells such as DP cells that are supplemented with PDGF also show restored inductive and proliferative capacity after being cultured in a 2D system. Moreover, FGF2 and PDGF exhibit a synergistic effect on the HF-inductive capacity of DP cells (Tomita et al., 2006; Kiso et al., 2015). A natural source containing FGF2 and PDGF is platelet-rich plasma (PRP), which is used in clinical practice for various conditions, including androgenic alopecia (Zheng et al., 2014; Gentile et al., 2015; Cook and Smith, 2018). When PRP is added to rodent DP cells, similar effects to those of exogenous FGF2 and PDGF are observed-increased proliferation and increased inductive capacity (Miao et al., 2013).

Hair follicle progenitor cells also lose trichogenous properties when 2D cultured *in vitro*; interestingly, however, they show increased migratory and proliferative capacity when cultured with media derived from DP cells (Fujie et al., 2001). Additionally, HF progenitor cells show hair-specific characteristics when cultured with DP cells (Roh et al., 2004; Leirós et al., 2012).

WNT10b is a powerful morphogen that is primarily involved in the induction phase of early HF development. Recent studies have shown that WNT10b not only promotes *in vitro* proliferation of DP cells through the WNT/β-catenin pathway but also restores the inductive capacity of DP cells when cultured as spheroids (Ouji et al., 2013; Wu et al., 2020). In addition to WNT signaling, BMPs are important when culturing DP cells to maintain their inductive capacity. Purified DP cells that lack BMPR1A fail to generate HFs when mixed with epithelial HF progenitor cells (Rendl et al., 2008). Moreover, this *in vitro* model showed that BMPR1A-ablated DP cells lose their DP signature genes due to a lack of BMP signaling. Losing DP signature genes indicates that BMP signaling is a crucial factor in the interaction between epithelial cells and DP to induce HF neogenesis *in vitro* (Rendl et al., 2008).

To mimic the necessary conditions for HF morphogenesis for tissue engineering purposes, it is important to consider the pleiotropic effects of TGF-β. When the concentration of the TGF-β1 morphogen is high in DP cells, keratinocyte growth in coculture is significantly suppressed (Inui et al., 2002). Moreover, when TGF-β1 is neutralized, keratinocyte growth is restored in a dose-dependent manner. It is thought that androgen-induced TGF-β1 is associated with alopecia development.

### *In vitro* Requirement of ECM Proteins for HF Neogenesis

Hair follicle neogenesis does not only rely on mixing different cell types with the correct concentration of exogenous morphogens. As mentioned before, cell–matrix interactions are very important in guiding progenitor cells toward (terminal) differentiation. A relatively simple and straightforward method to study cell–matrix interactions during HF neogenesis is to track the development of bioengineered hair germs *in vivo*. However, due to the complexity of the HF progenitor cell niche *in vivo*, these studies are quite challenging. To overcome these challenges, matrices have been designed to mimic the *in vivo* HF progenitor cell niche. The advantage of an artificial HF progenitor cell niche is that the composition and assembly of the components can be performed under standardized conditions. Therefore, the effects of other hair growth variables such as HF progenitor cells and morphogens can be more accurately monitored. Many natural and synthetic ECM scaffolds are commercially available, such as Matrigel^®^, as well as various forms of hydrogels and porous membranes that contain collagen I, and other components that are needed for ECM–cell interactions, such as chemical, mechanical, and physical cues, including focal adhesion via ECM-binding motifs (Figure 7; DuFort et al., 2011).

Because the main ECM component in the skin is collagen, most studies that track the development of neopapillae employ collagen I scaffolds (Abaci et al., 2018; Lalley and Boyce, 2019; Vahav et al., 2020). When rodent LGR6-positive keratinocytes are loaded in collagen I scaffolds, the keratinocytes are capable of inducing epithelization, hair growth, and angiogenesis in full-thickness wounds (Lough et al., 2016). Whether complete hairs, including nerve ends, can be generated is still under investigation. It is possible that besides morphogens, other ECM proteins, or scaffold molecules are needed. Fibrin is a natural scaffold originating from fibrinogen proteins, and is suitable for 3D culture of bioengineered hair germs because it contains sites for cellular adhesion (Xie et al., 2012; Xiao et al., 2017). Fibrin also plays an important role in angiogenesis, which can be advantageous for HF neogenesis (Zisch et al., 2001).

The composition of natural scaffolds is not constant and may contain impurities that can affect assay outcomes (Griffith and Swartz, 2006). Moreover, natural scaffolds tend to be vulnerable to biodegradability when used *in vitro*, and thus, their biomechanical properties may change during culture. Synthetic scaffolds are a good alternative to natural-based scaffolds for progenitor cell culture and bioengineered hair germs. The composition of these scaffolds is pre-defined, the mechanical properties can be easily manipulated, and the degradation rate is lesser. Moreover, in contrast to natural scaffolds, synthetic ECM proteins have little batch-to-batch composition variation, and have a reduced risk for pathogen contamination (Dawson et al., 2008; Ghasemi-Mobarakeh et al., 2015). It is important to note that Matrigel^®^ is mouse-derived and due to its batch-to-batch variation and undefined growth factors, it is neither safe nor practical for human studies (de Groot et al., 2020).

Polyethylene glycol diacrylate (PEGDA) is a synthetic polymer scaffold that is polymerized through UV crosslinking. PEGDA is a suitable scaffold for HF generation *in vitro* because of its adjustable mechanical properties and chemical composition, as well as the ability to engineer a suitable microenvironment through 3D microstructure fabrication (Xia and Whitesides, 1998; Zhu, 2010). Pan et al. (2013) showed that cells cultured in the PEGDA scaffold remain viable and are potentially useful for bioengineered hair germs *in vitro*. Other polymer scaffolds such as polycaprolactone, polylactide, and polyglycolide can be used, but clinical success is limited mainly due to the lack of focal adhesions and limited biological signals (Beumer et al., 1993; Dai et al., 2004). A relatively new method to create ECM scaffolds is self-assembling peptide hydrogels formed by RADA-16. One major advantage of RADA-16 over other synthetic scaffolds is the diameter of the microfibers, which is approximately 10 nm compared to 100 μm for synthetic microfibers (de Groot et al., 2020). When cultured with larger microfiber scaffolds, different cells can only adhere to one large fiber at a time, so the cells are effectively still in a 2D environment (de Groot et al., 2020).

RADA-16 has been found to enhance re-epithelization and increase wound closure *in vivo* (Schneider et al., 2008; Meng et al., 2009). A combination of RADA-16 with the binding motif RDG shows increased migration and proliferation of skin-derived precursors and enhanced expression of hair-induction signature genes such as AKP2 and BMP6 (Wang et al., 2016).

## Discussion

The aim of this review was to provide a better understanding of human HF development for future design of a human *in vitro* model, consisting of a full skin substitute with HFs, and its subsequent translation to clinical use. HF development relies on a perfectly synchronized spatiotemporal interplay between the HF progenitor cells, niche, and growth factors. Thus, timing–combining the right (progenitor) cell types, in the correct ratio, supported by the right growth factors, at the right time–is extremely important for obtaining a hair-containing skin equivalent. The literature indicates that during early HF development, the concentrations of growth factors and morphogens vary at specific time intervals, which is critical for further growth of the developing HF. The ECM molecules–mainly collagen 1 and laminin 511–do not seem to undergo many changes during this time.

During the early phases of creating a human HF organoid, early developmental proteins, including WNT10a, WNT10b, and DKK4, are determinative for the formation of a niche in which HF genesis can occur. Once the organoid is directed toward organogenesis, FGF20 is a key regulator for the formation of DC during the cytodifferentiation phase. Morphogens such as SHH, TGF-β, and PDGF are also crucial for further development of the HF.

We believe that to achieve hair growth in an *in vitro* model, a strict time- and nutrient-controlled protocol for each phase of HF development is crucial (Lee et al., 2020). To establish such an *in vitro* HF-neogenesis model, the use of microfluidic devices is essential as they can greatly facilitate spatiotemporal control of the cell microenvironment and allow gradual changes to the cellular milieu, e.g., growth factors and signaling molecules (Sutterby et al., 2020). Moreover, another aspect that needs to be considered is the establishment of standardized protocols for obtaining and culturing a purified human adult HF progenitor cell population, and how to transplant this HF progenitor cell pool into a relevant niche. Furthermore, the role of DP-derived spheroids is recognized to be important in tissue engineering strategies for establishing a hair-containing full skin equivalent. Therefore, investigating how to establish functional DP spheroids yielding HFs is essential. It is still unclear how and where human DP spheres can be inserted in the *in vitro* HF neogenesis program. In addition, the optimal ECM proteins, and/or the epidermal component needed for HF genesis from DP spheroids, warrants further research.

Aside from the above-mentioned conditions, increased research on vascularization of *in vitro* skin is warranted, as vascularization is crucial for driving the early HFs to further maturation (Abaci et al., 2018).

In the future, these models can perhaps be replaced by direct transplantation of particles containing a mixture of HF progenitor cells, morphogens, and ECM, with the potential to grow into HFs in human skin. *In vitro* models of HF neogenesis established using human cells offer great perspectives and are within reach; they will be of great use in the research of HF neogenesis *in vivo* and offer the opportunity to study patient-specific defects arising from signaling deregulation, such as alopecia and scar formation after burns.

## Author Contributions

All authors participated in the design of the study. SG and MH worked on the acquisition of the literature, interpretation of the data, and the writing of the manuscript. SG designed the figures. MU aided in interpreting the results and revising the manuscript critically for important expert judgment of the content. All authors contributed to the article and approved the submitted version.

## Conflict of Interest

The authors declare that the research was conducted in the absence of any commercial or financial relationships that could be construed as a potential conflict of interest.

## Abbreviations

BM: basement membrane
BMP: bone morphogenetic protein
CDP: CCAAT displacement protein
DC: dermal condensate
DKK: Dickkopf-related protein
DP: dermal papilla
ECM: extracellular matrix
EDA: ectodysplasin A
EDAR: ectodysplasin A receptor
FGF: fibroblast growth factor
FOX: forkhead box protein
HF: hair follicle
HFBSCs: hair follicle bulge stem cells
IRS: inner root sheet
Jag1: Jagged 1
LHX2: LIM/homeobox protein 2
LGR6: leucine-rich repeat-containing G-protein coupled receptor 6
MHC 1: major histocompatibility complex 1
NF- κ B: nuclear factor kappa-light-chain-enhancer of activated B cells
PDGF/PDGFA/PDGFRA: platelet-derived growth factor/platelet-derived growth factor subunit A/platelet-derived growth factor receptor A
PEGDA: polyethylene glycol diacrylate
RBP-Jk: recombination signal binding protein for immunoglobulin kappa J region
SOX: sex determining region Y-box
SHH: sonic hedgehog
TGF: transforming growth factor
WNT: wingless and Int-1.
